# First-principles studies of the SCl_2_ adsorption on the doped boron phosphide monolayer

**DOI:** 10.1007/s00894-025-06333-8

**Published:** 2025-03-08

**Authors:** Akari Narayama Sosa, Sandra Esteban Gómez, Juan Carlos Moreno Hernández, Dolores García Toral, Gregorio Hernández Cocoletzi

**Affiliations:** 1 Benemérita Universidad Autónoma de Puebla, Instituto de Física, Ciudad Universitaria, Av. San Claudio y Blvd 18 sur, Col. San Manuel, Puebla, 72570 México; 2 Benemérita Universidad Autónoma de Puebla, Facultad de Ingeniería Química, Av. San Claudio y 18 Sur S/N, San Manuel, Puebla, 72570 México; 3 On Sabbatical Leave at Instituto Nacional de Astrofisica, Optica y Electrónica, Tonantzintla, Puebla, México

**Keywords:** 2D materials, SCl_2_, Boron phosphide, Adsorption, Doping, Detection

## Abstract

**Context:**

Sulfur dichloride (SCl_2_) molecules form a harmful substance; however, it is widely used in the industry as insecticide and in organic synthesis. In contact with water, these molecules produce other toxic and corrosive gases. Therefore, it is important to remove them from the environment. In this work, we have studied the boron phosphide (BP) monolayer (ML) doped with metal atoms to be considered as a sensor material for the detection of sulfur dichloride (SCl_2_) molecules. Studies are done by applying the density functional theory (DFT) according to the PWscf code of the Quantum ESPRESSO, using the projector-augmented-wave (PAW) method within the framework of the generalized gradient approximation (GGA) with the PBE parameterization. The results obtained indicate weak interactions between the SCl_2_ molecule and the pristine BP monolayer. However, after metal-doping (with atoms of: Ga, In, N and As) the interactions between the SCl_2_ molecule and the ML was increased, as expected. Parameters such as the adsorption energy (E_ad_), work function (Ф), Bandgaps (E_g_), recovery time (τ), electronegativity (χ) and chemical potential (μ) have been analyzed. The results suggest that the metal-doped BP monolayer may be a promising sensing material for gas sensor devices to detect SCl_2_ molecules.

**Methods:**

The SCl_2_-metal-doped BP ML has been investigated using DFT calculations as implemented in the PWscf code of the Quantum ESPRESSO, and using PAW pseudopotential within the framework of the GGA-PBE and energy cutoff of 40Ry. The force components were smaller than 0.05 eV/Å and the Grimme-D2 scheme was considered. The Brillouin zone was sampled using a Monkhorst–Pack grid of 5 × 5 × 1 and 17 × 17 × 1 k-points for structural relaxations and electronic-properties calculations.

**Supplementary Information:**

The online version contains supplementary material available at 10.1007/s00894-025-06333-8.

## Introduction

Pollution is caused by harmful components, such as chemical, physical or biological which may be present in the environment, and are dangerous to living beings, including humans. The main sources of environmental pollution are those derived from the human activity and natural sources, that have caused the phenomena of climate change and the global temperatures rise [1]. In order to evaluate the concentrations of pollutants in the environment and thereby adopt strategies to reduce their emission, it is necessary to fabricate sensor devices to detect minimal changes in the concentration of pollutants. In this regard, gas sensors play a crucial role in various fields, such as environmental monitoring and medical engineering. These toxic gas detector devices have emerged especially from small-scale materials [2]. Therefore, the search for materials to improve gas sensors devices is of vital importance [3, 4]. In this context, two-dimensional (2D) materials are gaining a great importance in the nanotechnology because these systems offer a great potential for the fabrication of gas sensing devices, due to the large surface-to-volume ratio and unique properties (electrical, optical and mechanical properties) [5, 6]. A 2D materials such as graphene, is considered as an ideal candidate because of its high electron mobility (10^5^ cm^2^ /V s) [7], and other characteristics that make it a promising material in the field of gas sensor [8–11]. Other 2D materials, such as the nitrides (GaN, BN, AlN) especially the x-nitride nanoribbons reported by Rai and Jha [12–17], silicene [18, 19], arsenene [20], stanine [21], SnSe [22], borophene [23], germanene [24, 25], and siligene [26] are possible sensitive materials that may be applied in gas sensor devices. However, several materials (silicene and graphene) have zero gap, which limits their direct application in nanoelectronics devices. Therefore, finding new 2D materials, that exhibit an appropriate bandgap energy and also good structural stability, is critical for their potential applications. Hexagonal boron nitride (h-BN) [27] exhibits a wide bandgap with a potential application as a dielectric material. Black phosphorene (BlackP) [28–32], and blue phosphorene [33, 34] are suitable for the incorporation of metals (alkaline, alkaline earth, non-metallic, transition and noble) to modify the phosphorene semiconductor behavior. Currently, interest has been paid on the boron-phosphide monolayer, provided that this may be applied in the nanoelectronics industry. This material has a good structural and mechanical stability, exhibits Hall effect [35–37], high carrier mobility (~ 10^4^ cm^2^/V s), and a moderate direct bandgap between 0.9 and 1.37 eV [38]. Although the BP single crystal was synthesized a long time ago [39], the BP ML has not been synthesized yet, nevertheless the BP films growth by chemical vapor deposition, as well as prepared by co-evaporation of B and P on glass and Si (100) substrates have been reported. [40, 41]. Furthermore, boron phosphide has been studied theoretically, including phononic calculations, which show the structure stability [27, 42]. On the other hand, it has been shown that the alteration of the surface using special atoms may improve the interaction of the monolayers with different types of molecules. Hence, the hexagonal boron phosphide monolayer has been investigated taking into account dopant atoms, in a similar fashion as reported by Onat et al. [43]. In addition, there are other works that report the effects of doping and the functionalization of the boron phosphide monolayer [44–48] that together with the reported works of h-BN, and black and blue phosphorene may be an alternative to the present work. Besides, the effects of vacancies and doping on the electronic properties have also been studied in graphene [49], phosphorene [50], and boron nitride (BN) [51].

Motivated by the good results reported of the doping effects in several works and with the purpose of detecting polluting gases, such as sulfur dichloride (SCl_2_) [52–54], we have investigated the capacity of the metal-doped BP ML for the adsorption of the SCl_2_ molecules. Our studies include the substitution of the pristine BP ML by the metal-doped BP layer to improve the absorption of the molecule, and modify the structural and electronic properties, as well as the variation of the adsorption (substitution) energies, work function and recovery time of the systems at hand.

## Computational details

The structural and electronic properties of the SCl_2_ adsorption on the pristine and metal-doped BP-(4 × 4) ML are investigated by first-principles total energy calculation using the density functional theory (DFT), as implemented in the PWscf code of the Quantum ESPRESSO package [55]. The exchange and correlation energies are modeled within the generalized gradient approximation (GGA) as proposed by Perdew-Burke-Ernzerhof (PBE) [56]. The electron–ion interactions are treated by the projector-augmented-wave (PAW) potentials [57, 58]. Electronic states are expanded in plane waves with an energy cutoff of 40 Ry. The geometry optimization is achieved when force components are smaller than 0.05 eV/Å and we have also considered the Grimme-D2 scheme of dispersion correction to include van der Waals interactions in the systems [59].

The Brillouin zone integration has been done using a Methfessel Paxton [60] smearing of 0.05 Ry and a k-points grid of 5 × 5 × 1 has been employed to sample the Brillouin zone according to the Monkhorst–Pack scheme [61]. The supercell has a 4 × 4 surface periodicity. For all supercells, we have also considered a vacuum space of 23 Å along the perpendicular direction to the monolayer. The k-points grid were increased to 17 × 17 × 1 to calculate the electronic properties.

## Results and discussion

**Fig. 1:**
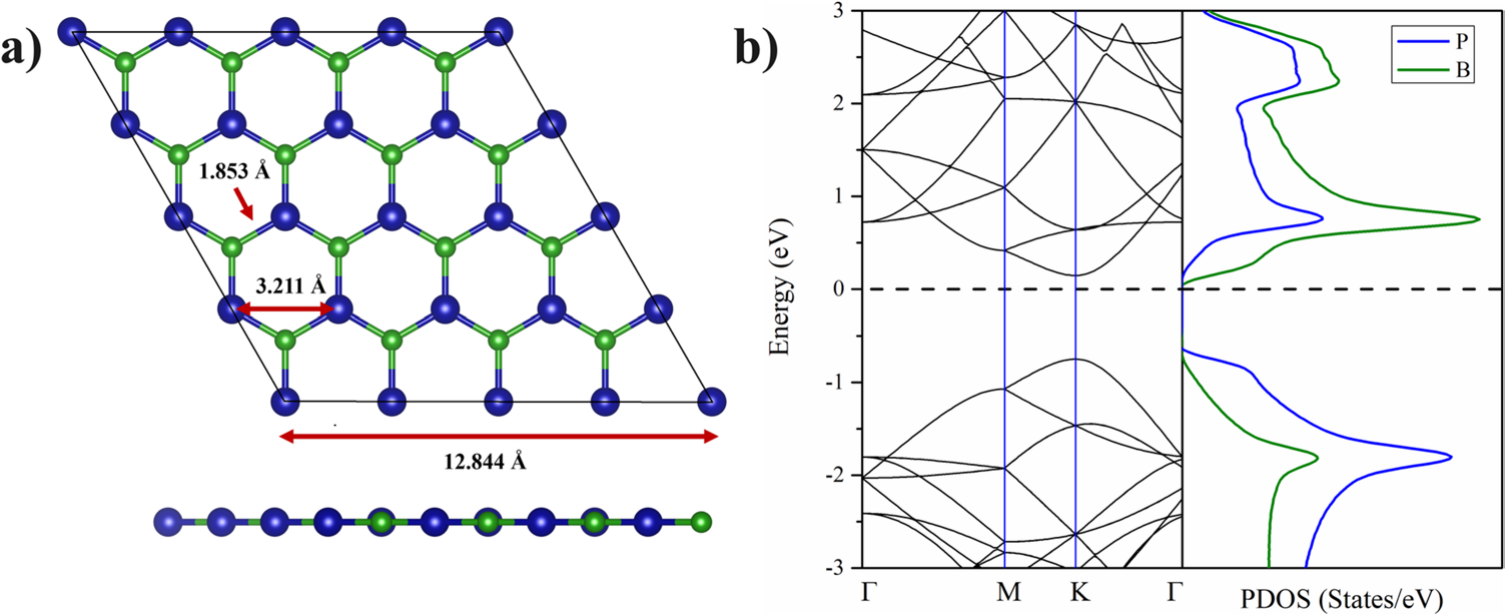
**a**) Top and side view for the optimized structures of 32 atom BP ML, **b**) band structure and density of states of the BP ML

Figure 1a displays the optimized structure (top and side views) of the 4 × 4 BP monolayer**.** The calculated lattice constant of the hexagonal BP ML is a = b = 3.21 Å, in agreement with previous theoretical results [27]. The B-P bond length is 1.853 Å, which is larger than the B-N bond length of 1.45 Å in a BN monolayer [62], with elongation being attributed to the larger atomic radius of P with respect to the N atom. Figure 1b displays the electronic band structure and the density of state of the 4 × 4 BP ML, which indicates that the BP ML is a semiconductor with a direct bandgap of 0.896 eV, in agreement with previous results [27, 38]. The BP monolayer has a graphene-like planar structure as induced by the sp^2^ orbitals hybridization [9].

**Fig. 2:**
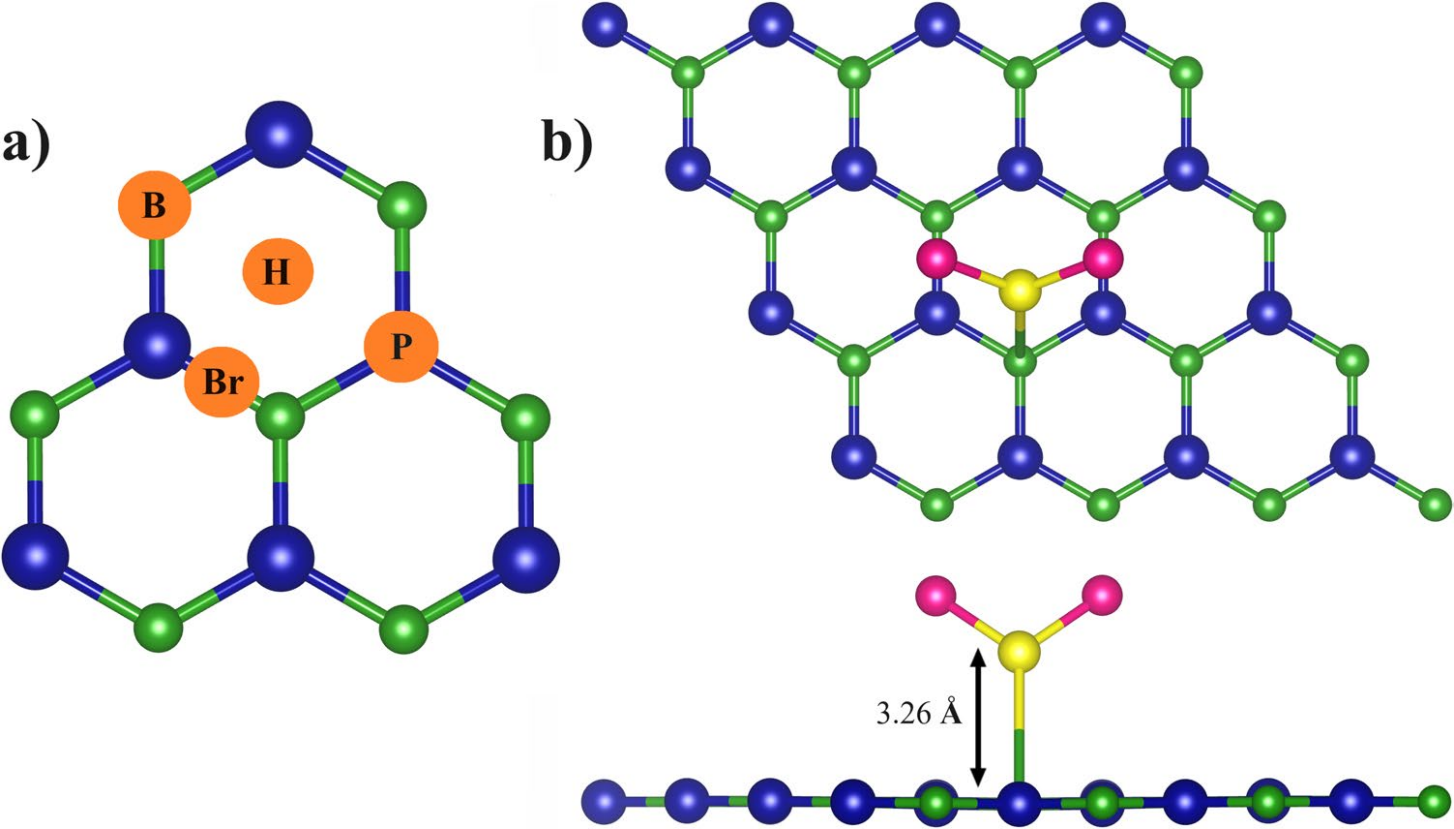
** a**) Adsorption sites for molecule on BP monolayer and **b**) Top and side views of SCl_2_ adoption on 32 atoms BP monolayer

The SCl_2_ molecule adsorption onto the pristine boron phosphide and metal-doped BP monolayer has been examined. First, we have studied the adsorption of the molecule on the pristine BP monolayer surface (see Fig. 2b). For this purpose, we have explored different orientations of the molecule at different high-symmetry sites, see (Fig. 2a). The high-symmetry sites are: Bridge (B), hollow (H), on top of B (B) and on top of P (P).

To determine the molecule adsorption energies, we have used the binding energy definition:

1 $${E}_{b}=\left(E\left(BP+M\right)-E\left(BP\right)-N{*}E\left(M\right)\right)/N$$

where E(BP) is the total energy of the BP pristine monolayer, E(M) is the energy of the isolated molecule, E(BP + M) is the energy of the optimized structure after adsorption and N is the number of dopant atoms.

The strongest interaction was obtained when the molecule was vertically oriented with the S atom bound to the B atom, with an interacting distance of 3.26 Å and an adsorption energy of −0.28 eV (Fig. 2b). The SCl_2_ molecule adsorption exhibits a weak interaction with the monolayer which is corroborated with the partial density of state. The system exhibits a bandgap of 0.904 eV (Figure S1, Supplementary Information).

Doping the monolayer, with different metal atoms, was done to enhance the molecule adsorption. When the dopant is introduced in the BP monolayer, two possible cases are studied: the substitution of a B or P atom by the dopant (Ga, In, N and As). The substitution energy was calculated using the following definition

2 $$E_s=E\left(BP\right)+E\left(X\right)-E\left(BP+X\right)-E\left(B/P\right)$$

where E(BP) is the total energy of the pristine BP monolayer, E(X) is the energy of one dopant atom, E(BP + X) is the energy of the structure with a dopant atom at B/P sites and E(B/P) refers to the single atom energy of B or P that is substituted, respectively.

**Fig. 3:**
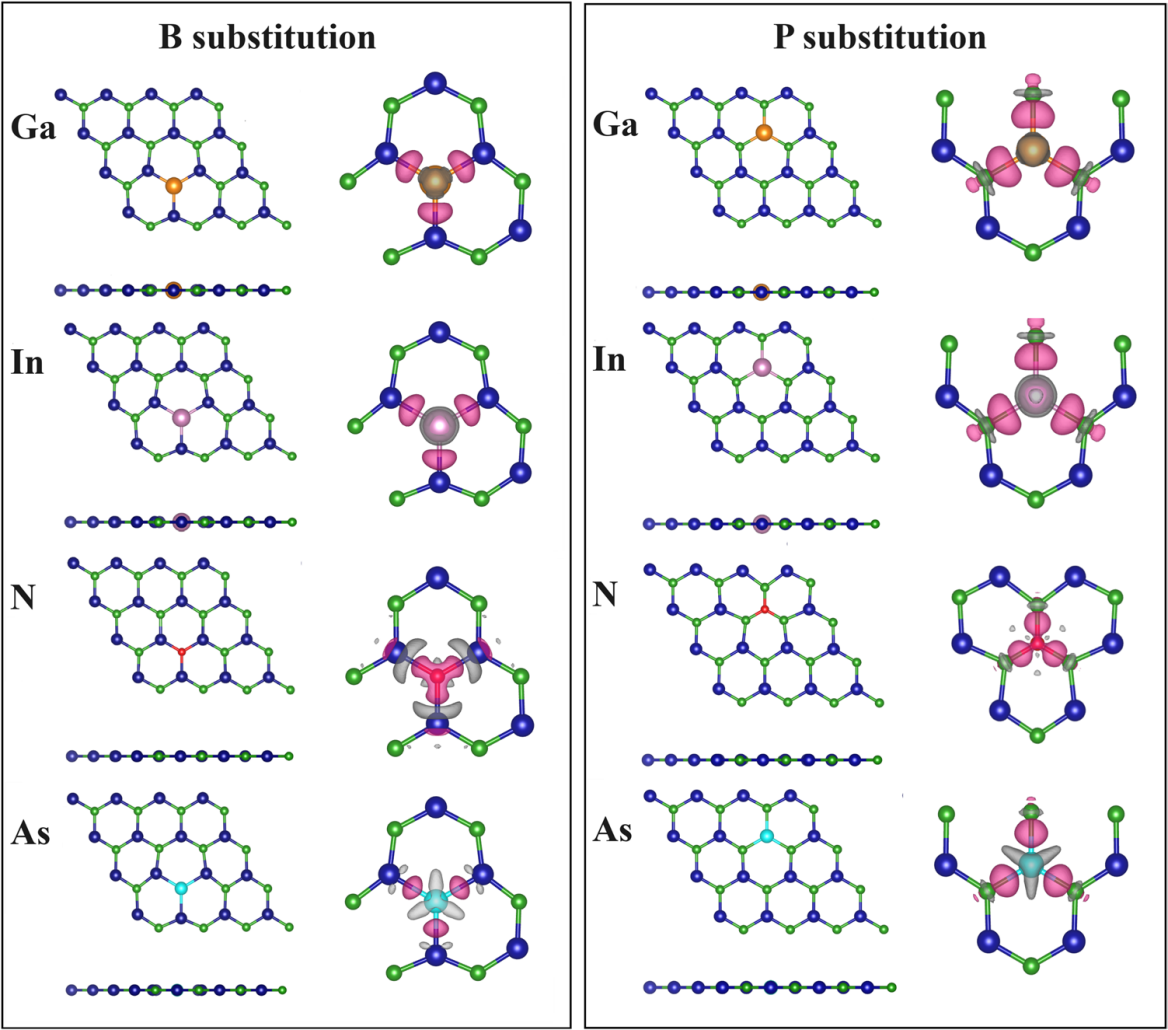
Optimized structures and charge density difference (CDD) isosurfaces of the doped BP monolayer with **a**) Gallium, **b**) Indium, **c**) Nitrogen and **d**) Arsenic. The left and right schemes represent the B substitution and P substitution, respectively. Pink and grey regions refer to charge accumulation and depletion, respectively

Figure 3 shows the optimized structures after metal atoms incorporation (BP-Ga, BP-In, BP-N and BP-As), left side is for the atomic structure and right side is for the charge density difference. Lattice distortions after substitutions were only observed at the vicinities of the dopant atoms.

The charge transfer on each individual atom induced by the dopant atom was determined with the Bader charge analysis as shown in Table 1 and Fig. 3. A small amount of charge (δ) has been transferred to the N dopant atom. In both cases of substitution, the N atom acts as a charge acceptor. Specifically, the largest charge transfer occurs when the B atom is substituted by N accept a charge of −0.69e from the BP monolayer. Ga, In and As atoms show a charge depletion, with an accumulation charge on the BP monolayer, resulting in a positive charge (δ). This change can be attributed to the relatively low electronegativity (χ) of these three elements compared to the atoms that form the pristine BP monolayer. The difference in the charge transfer is also reflected in the charge density differences shown in Fig. 3, which shows the interaction between the dopant atoms and the BP monolayer.

**Table 1:** Substitution Energies (E_s_), bonding lengths to nearest neighbor B and P atoms (D_b_, D_p_), Charges Transfer on dopant atoms (δ) Bandgap Energy (E_g_), Work Function (Ф), Chemical Potential (μ) and Electronegativity (χ) of the metal-doped BP monolayer

Substitute	Properties	Ga	In	N	As
B	E_s_ (eV)	−5.86	−7.70	−1.32	−6.17
	D_b_ (Å)	3.31	3.34	3.16	3.30
	D_p _(Å)	2.13	2.24	1.80	2.15
	E_*g*_ (eV)	0.90	0.88	0.43	–
	δ(e)	0.21	0.43	−0.69	0.14
	Ф (eV)	2.71	2.69	2.70	2.50
	μ (eV)	−0.45	−0.44	−0.21	–
	χ (eV)	0.45	0.44	0.21	–
P	E_s_ (eV)	−5.52	−6.93	3.18	−1.57
	D_b_ (Å)	2.01	2.00	2.11	1.54
	D_p_(Å)	3.25	3.25	3.29	3.10
	E_*g*_ (eV)	0.56	0.46	1.03	0.87
	*δ(e)*	0.57	0.74	−0.31	0.61
	Ф (eV)	3.48	3.28	2.76	2.76
	μ (eV)	−0.28	−0.23	−0.51	−0.44
	χ (eV)	0.28	0.23	0.51	0.44

Substitution energies (E_s_), bond lengths to nearest neighbor B and P atoms (D_b_, D_p_), energy (E_g_) bandgaps, charge excesses on dopant atoms (δ), Work Function (Ф), chemical potential (μ) and electronegativity (χ) are listed in Table 1**.** When an B or P atom is replaced by an X atom, the B-P (1.853 Å) and P-P (3.21 Å) bond lengths change slightly. The bond distance in almost all systems increases, while it slightly decreases when the dopant atom N replaces the B (a = 1.80 Å) and when As substitutes the P atom (a = 1.54 Å). Analyzing the substitution energies E_S_ in Table 1, only the substitution of P by N turns out to be positive, indicating an exothermic reaction, and all other cases require energy. For all other elements, E_S_ is negative and the value increases as both B and P is substituted by atoms belonging to the same column of the periodic table.

**Fig. 4:**
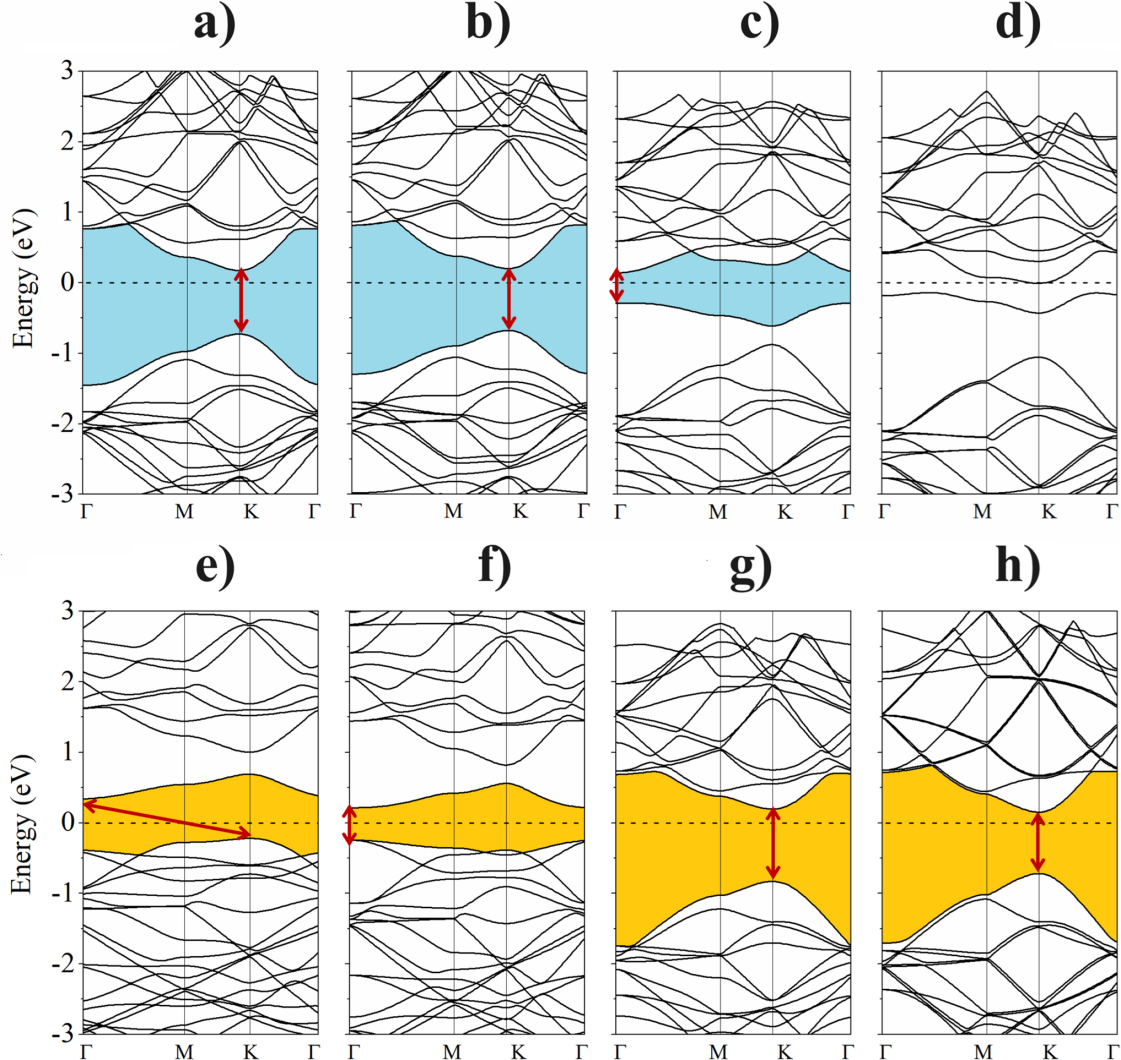
Electronic band structures of metal-doped BP ML, **a**) Ga, **b**) In, **c**) N and **d**) As for the substituted B atom, and **e**) Ga, **f**) In, **g**) N and **h**) As for the substituted P atom

Figure 4 shows the electronic band structures of the 4 × 4 metal-doped BP monolayer. The bandgap in most cases decreases after the replacement of the B and P atoms by the dopant atoms, it even vanishes, as in the case of the incorporation of As in the B site. However, in contrast the only case where the bandgap increase is for the N doping at the P site. Furthermore, we note that the unique case with an indirect bandgap of 0.9 eV is that when Ga replaces P. Figures 4a to d represent the electronic band structures when the B atom was substituted and Figs. 4 e to h show the electronic band structures when the P atom was substituted. The rest of the electronic structure information of dopant atoms is summarized in Table 1.

(Figure S2, Supplementary Information) In the partial density of the state (PDOS), we find the following results: When the B atom is replaced by Ga and In elements, no additional states are induced. Only small effects were encountered in the valence and conduction bands and, E_g_ of the pristine BP is preserved. On the other hand, substitutions with N and As yield an additional electron state, when the As is incorporated the system becomes metallic. For the case of the N dopant, the systems become n-type semiconductors with the generation of two states localized close the Fermi level, however, only one state is occupied. When a P atom is substituted by Ga or In elements, the PDOS indicates that both dopant atoms induce electronic states into the valence and conduction bands. Meanwhile, substitutions with N and As no additional states are generated.

Additionally, two chemical reactivity descriptors are determined directly from the DFT calculations, such as the electronegativity (χ) and the chemical potential (μ), as follows [63, 64].

3 $$\mu =-\chi = \frac{IP+EA}{2}=\frac{\varepsilon_{HOMO}+\varepsilon_{LUMO}}{2}$$

As it is noted, the electronegativity is the negative of the chemical potential (the Lagrange multiplier for the normalization constraint), which was also formulated by Parr [65, 66]. To obtain these two reactivity indices, the ionization potential (IP) and electron affinity (EA) are firstly obtained semi-quantitatively [64]. Using the results of the highest occupied molecular orbital energy (HOMO) and the lowest unoccupied molecular orbital energy (LUMO) [67] the following equations are obtained:

4 $$IP = {\varepsilon }_{HOMO}, EA = - {\varepsilon }_{LUMO}$$

Therefore, the chemical reactivity of the different doped BP monolayers was analyzed from the results of the chemical potential and electronegativity. A small chemical potential indicates a large E_g_, since they are usually inversely proportional. On the other hand, a small electronegativity would indicate a charge transfer by the doping atoms, as indicated in Table 1.

Once the calculations of the BP-doped monolayer were carried out, the adsorption of the molecule (SCl_2_) on the surface was studied. The adsorption takes place with different orientations at high symmetry sites. The bond angles of the adsorbed SCl_2_ molecule on the doped BP monolayer corresponding to the most stable configurations are presented in (Table S1, Supplementary Information). The Cl_1_ − S − Cl_2_ bond angle of the SCl_2_ molecule before optimization on the monolayers is 104.99°. The optimized Cl_1_ − S − Cl_2_ bond angle in the pristine layer is 104.23°. The bond angles except for the Ga-doped system for B substitution appear slightly smaller as compared to the pristine case. The molecule adsorption on the doped monolayer induces a variation in the Cl_1_ − S − Cl_2_ molecule with a significant dependence on the doping element. In general, the bond length and angle variations induced by the doping element in the monolayer are still within a reasonable range.

To be a promising sensing material for the adsorption of molecules, a relatively strong adsorption energy with the metal-doped monolayer is necessary. The adsorption energies (E_ad_), bandgap energy (E_g_), charge transfer on the molecule (δ), adsorption distance (DM-BP) from the nearest atom of the gas molecule to the atom of the metal-doped BP monolayer, work function (Ф), chemical potential (μ), electronegativity (χ) and recovery time (τ) are listed in Table 2. When the adsorption energies E_ad_ are analyzed, we note that in most cases the adsorption of the molecule increases, as a function of the dopants, showing that the highest adsorption energies are for the As and In doped systems with the incorporation being at the B site.

**Table 2:** Adsorption Energy (E_ad_), Adsorption Distance (D_M-BP_), Bandgap Energy (E_g_), Charge Transfer on the molecule (*δ*), Work Function (Ф), Chemical Potential (μ), Electronegativity (χ) and Recovery Time (τ) of the molecule metal-doped BP monolayer

Substitute	Properties	Ga-SCl_2_	In-SCl_2_	N-SCl_2_	As-SCl_2_
B	E_ad_ (eV)	−1.39	−2.39	−0.67	−3.09
	D_M-BP_ (Å)	2.71	2.27	2.34	2.69
	E_*g*_ (eV)	1.17	0.62	0.60	0.82
	*δ(e)*	−0.76	−1.19	−1.16	−1.12
	Ф (eV)	2.69	3.18	3.17	3.08
	μ (eV)	−0.58	−0.31	−0.30	−0.41
	χ (eV)	0.58	0.31	0.30	0.41
	τ (300 K)	7.12 × 10^3^ years	4.48 × 10^20^ years	1.8 × 10^–1^ sec	9.41 × 10^34^ years
P	E_ad_ (eV)	−0.79	−1.18	−0.32	−0.29
	D_M-BP_ (Å)	2.44	2.60	3.26	3.26
	E_*g*_ (eV)	0.74	0.68	1.04	0.89
	*δ(e)*	−0.67	−0.72	−0.82	−0.82
	Ф(eV)	3.27	3.12	2.61	2.60
	μ (eV)	−0.37	−0.34	−0.52	−0.45
	χ (eV)	0.37	0.34	0.52	0.45
	τ (300 K)	18.68 sec	2.11 years	2.38 × 10^–7^ sec	7.44 × 10^–8^ sec

The electronic structures of the complete systems changed and the charge was distributed between the molecule and the monolayer. In Table 2, we can note the amount of charge yielded by the gas molecule. The charge density difference (CDD) was calculated and the results are exhibited in (Figure S3, Supplementary Information). An amount of transferred charge was found in the SCl_2_ molecule, which acts as an electron acceptor, the highest charge transfer occurs when the B atom is substituted. The results of the chemical potential and electronegativity of the SCl_2_ molecule adsorbed on the doped BP monolayer, indicate that the chemical potential is not only inversely proportional to the E_g_, but also predicts the trend of the adsorption energy, so a small chemical potential indicates a higher adsorption energy.

**Fig. 5:**
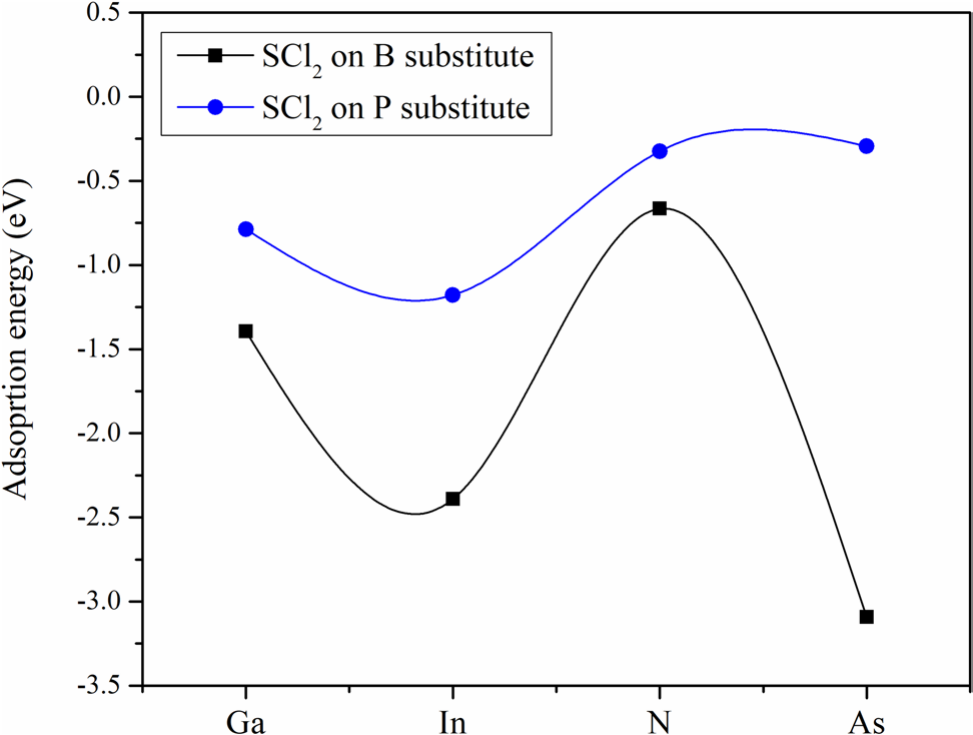
Comparison of the adsorption energies of the SCl_2_ on metal-doped BP ML

Figure 5 shows a comparison of the SCl_2_ adsorption energies when adsorbed on the metal-doped BP monolayers for both the B and P substitutions. Although the E_ad_ are very different, the results suggest that the SCl_2_ molecule is adsorbed more strongly on the doped BP, except for the case with As-doped ML at the P site, which has an adsorption energy of −0.29 eV. In all cases, an increased in E_ad_ is obtained with respect to the pristine system (−0.28 eV). The adsorption energy is stronger when the substituted atom is B except when the N is the dopant, this can be attributed to the atomic size of N and B (see Fig. 5).

**Fig. 6:**
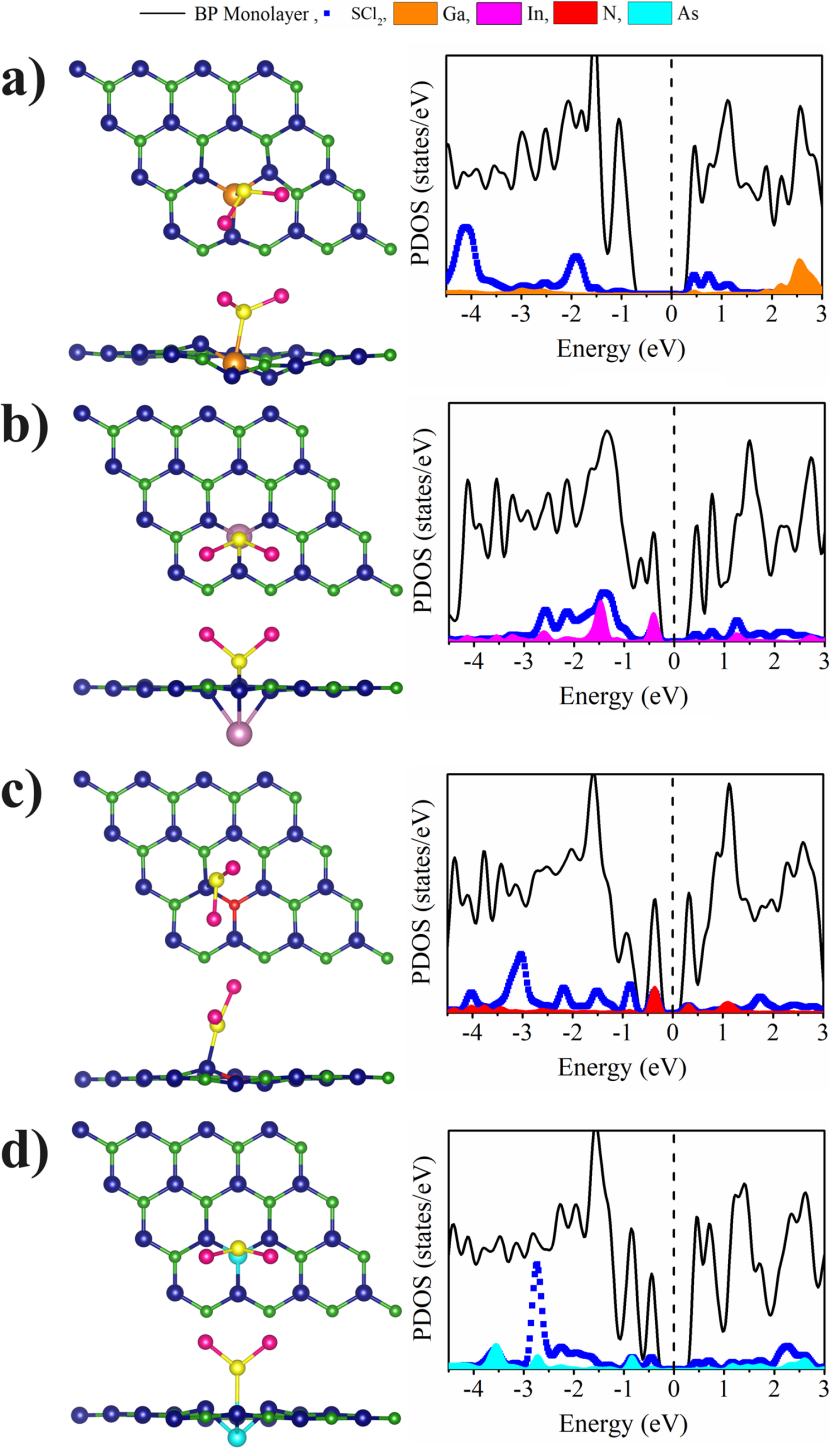
Optimized structures (top and side view) and partial density of electronic states of SCl_2_ molecule adsorbed on metal-doped BP ML for the B substituted by: **a**) Ga, **b**) In, **c**) N and **d**) As atoms. The Fermi levels are shifted to zero (black dashed lines)

**Fig. 7:**
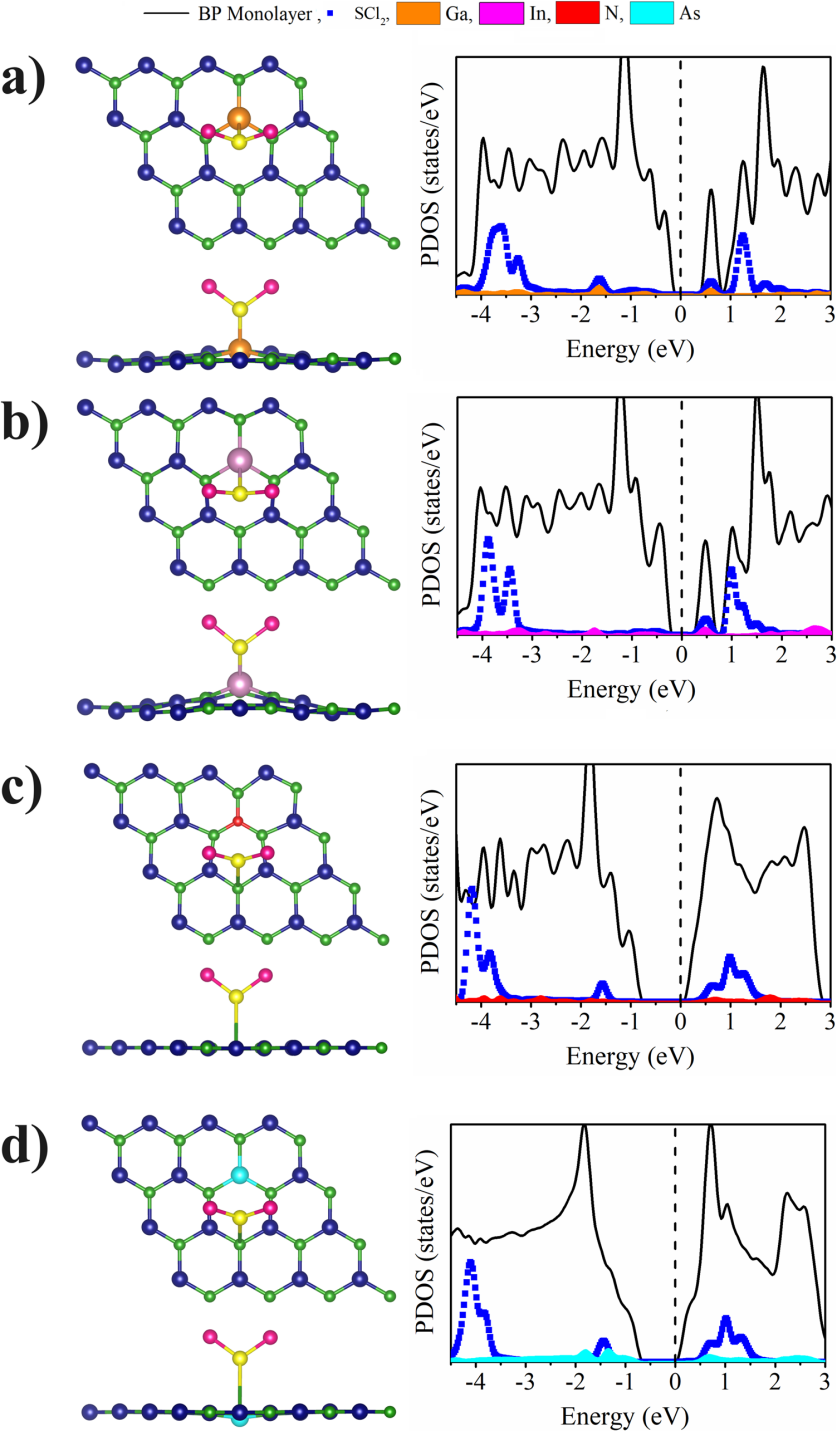
Optimized structures (top and side view) and partial density of states of SCl_2_ molecule adsorbed on metal-doped BP ML  for the P substituted by: **a**) Ga, **b**) In, **c**) N and **d**) As atoms. The Fermi levels are shifted to zero (black dashed lines)

Figure S4 (Supplementary Information) shows the electronic band structure of the metal-doped BP ML with the SCl_2_ adsorbed. When the B is substituted by As, the bandgap is of the order of 0.62 eV, and when the ML is doped with In the bandgap is of 0.62 eV. In general, the SCl_2_ molecule adsorption increases the bandgap except when the In is incorporated at the B site and a new indirect transition is obtained in the In doped case in the P substitution. On the other hand, it can be noted the presence of states corresponding to the SCl_2_ molecule and the metals near the valence and conduction bands, as shown in Figs. 6 and 7. The hybridization of the SCl_2_ molecule states with those of the metal atom (Ga, In and As) is also obtained close to the Fermi level, in the incorporation at the B atom site. When the P atom was substituted, the hybridization takes place with the Ga and In state, which exhibit the highest E_ad_ values. On the other hand, substitution with N at the boron site (Fig. 6) and substitution with N and As at the phosphorus site (Fig. 7) do not show hybridization with the states of the SCl_2_ molecule, and at the same time these systems exhibit the smallest E_ad_ values.

To explore the efficiency of the sensor devices, we have calculated the work function Ф of the gas molecule before and after the absorption, here Ф represents the minimum energy required to remove electrons from the surface of a solid material and is defined as:

5 $$\Phi ={E}_{vac}-{E}_{Fermi}$$

where *E*_*vac*_ and *E*_*Fermi*_ are the vacuum energy and the Fermi level, respectively [24, 68, 69].

The work function of the pristine BP monolayer (2.78 eV) and that of the BP monolayer after the SCl_2_ adsorption (2.60 eV) shows a decrease. In Tables 1 and 2**,** the results of the work functions of the metal-doped monolayer before and after the SCl_2_ adsorption are summarized. The decrease in the Ф indicates an increase in the electron transfer from the material, which leads to an improvement in the performance. It can be noted that different doping leads to adjustable work functions. It is apparent that the work function of doped monolayer is modified by the SCl_2_ adsorption. The corresponding changes in the work function (ΔΦ) of the metal-doped ML after the SCl_2_ adsorption, are −0.02, 0.49, 0.47 and 0.58 eV in the B substitution and −0.21, −0.16, −0.15 and −0.16 eV in the P substitution, as Ga, In, N and As are the dopant, respectively. Note that the largest changes in the work function are for the Ga-doped case at the B site and for all cases in the P substitution, indicating the possibility of gas sensor applications.

The recovery time can also be used to determine a good performance of a gas sensor device [70, 71]. Therefore, it is important to calculate the recovery time and get a small value. The following equation is used to calculate the recovery time as a function of the temperature

6 $$\tau ={A}^{-1}{E}^{\left(\frac{{E}_{ad}}{{k}_{B}T}\right)}$$

where A is a frequency factor (10^12^ s^−1^) [72, 73], k_B_ is the Botlzmann constant, T is the temperature, and finally, E_ad_ (eV) is the adsorption energy. The results of $\tau$ are listed in Table 2. Where we note that the recovery time of the SCl_2_ molecule adsorbed on the pristine BP monolayer was of 5.06 × 10^–8^ s, which show that the BP monolayer has rapid recovery time. For the cases where the boron atom is replaced by the Ga, In and As atoms and for the case where the P atom is replaced by the In atom, we have a very long recovery time, which makes the molecule desorption a very difficult task and the reuse of the metal-doped BP monolayer as a sensor. However, for the other cases where the boron atom is substituted by the N atom and the P atom is replaced by the Ga, N and As atoms, the results show a considerable recovery time (in seconds), specially where the P atom is replaced. They may possess potential applications as material for sensor devices. The results also indicate that the presence of the SCl_2_ molecule may strongly limit the use of the metal doped BP monolayer as a gas sensor for cases where the recovery time is quite long. So, it can probably be used as a trapping material of dangerous molecule. As expected, desorption times depend on the adsorption energy and temperature. If the temperature increases the recovery time may reduce, so temperature plays an important role in the development of a sensor devices.

**Fig. 8:**
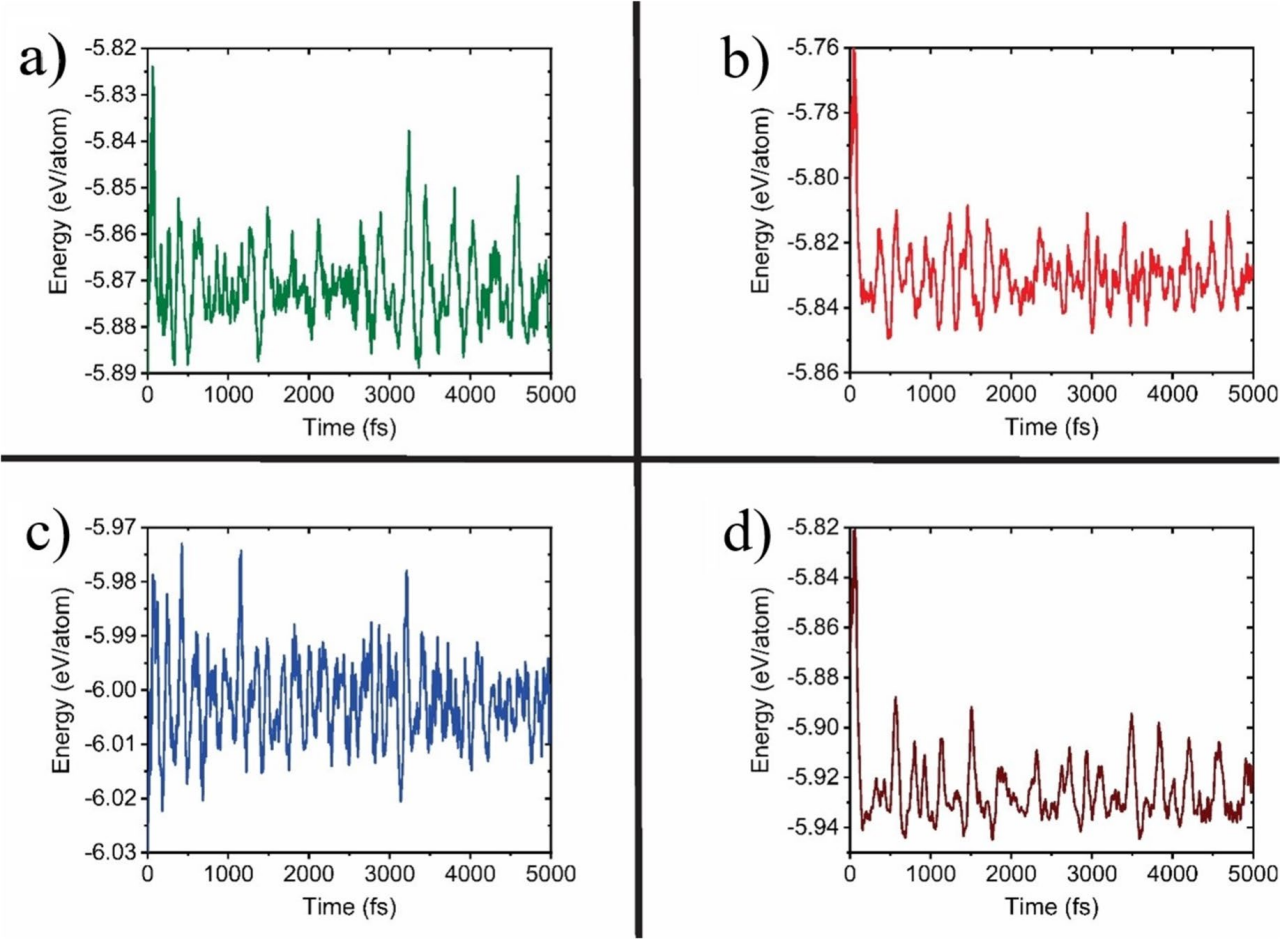
The MD simulation energies to examine the stability of metal-doped BP ML system at 300 K, for the P substituted by: **a**) Ga, **b**) In, **c**) N and **d**) As atoms

To stress on the structure stability, *ab-initio* molecular dynamics calculations were developed to examine the thermal stability of the most stable structures at room temperature. The Vienna Ab initio simulation package (VASP) software [74] with a fixed particle number, volume, and temperature (NVT) was used. The Nose–Hoover [75] thermostat method is employed at 300 K for a total 5 ps with a time interval of 2 fs. The variation in energy is quite small in all cases, and according to the substitution the systems that require less energy are those for the P atom substitution (Fig. 8). The behavior of the total energy as a function of time and temperature indicates that the metal-doped BP monolayers are stable at room temperature. Mention must be done that here we only report the MD calculation when the P atom is substituted, however, more MD energies images of the doped BP ML system are reported in (Figure S5 of the Supplementary Information), when the B atom is substituted.

## Conclusions

In this work, the interactions of the SCl_2_ molecule with the pristine and metal-doped BP monolayer (ML) have been studied through first-principles calculations based on the density functional theory (DFT) to search for new materials to be applied in the fabrication of sensor devices. For the pristine BP monolayer, we found that the SCl_2_ molecule was physisorbed. To improve its reactivity and enhance the interaction with the molecule, the BP monolayer was doped with metallic atoms (Ga, In, N and As). The results indicate that, as expected, the doping enhances the interaction between the SCl_2_ molecules and the boron phosphide ML, increasing the adsorption energy and changing the energy gap. However, a slight deformation can also be observed on the part of the systems that does not affect the interaction of the systems. The electronic properties display very different features, in most cases the energy bandgap decreases and the PDOS shows the hybridization of the molecule and metal-doped BP states. The corresponding changes in the work function (ΔΦ) when SCl_2_ is adsorbed on the dopants; Ga, In, N and As, incorporated at the B site in the BP monolayer were −0.02, 0.49, 0.47 and 0.58 eV, respectively. When the P is substituted in the ML, the changes in the work function were −0.21, −0.16, −0.15 and −0.16 eV for Ga, In, N and As, respectively. Results of the recovery time of SCl_2_-doped BP monolayer had the following tendency τ(In) > τ(Ga) > τ(N) > τ(As) for the P substitution, and τ(As) > τ(In) > τ(Ga) > τ(N) for the B substitution. These yielding a suitable material for gas sensor devices. These results indicate that the metal-doped BP monolayer can be used as a gas sensor to detect SCl_2_ in the atmosphere.

## Supplementary Information

Below is the link to the electronic supplementary material.

ESM 1 (DOCX 2.45 MB)

## Data Availability

No datasets were generated or analysed during the current study.
