# Supplementary material for: First-principles studies of the SCl2 adsorption on the doped boron phosphide monolayer
Source: J Mol Model. 2025 Mar 8;31(4):111. doi: 10.1007/s00894-025-06333-8 (PMC11890252; doi:10.1007/s00894-025-06333-8)
Supplement: Supplementary file 1 — (DOCX 2.45 MB) [file 894_2025_6333_MOESM1_ESM.docx]

Supplementary material

**First-Principles Studies of the SCl_2_ adsorption on the doped boron phosphide monolayer**

Akari Narayama Sosa^1^*, Sandra Esteban Gómez ^1^, Juan Carlos Moreno Hernández^1^, Dolores García Toral^2^, Gregorio Hernández Cocoletzi^1^

*Benemérita Universidad Autónoma de Puebla, Instituto de Física, Av. San Claudio y Blvd 18 sur, Col. San Manuel, Ciudad Universitaria, Puebla 72570, México*

*^2^Benemérita Universidad Autónoma de Puebla, Facultad de Ingeniería Química, Av. San Claudio y 18 Sur S/N, San Manuel, Puebla 72570, México.*

*^3^On sabbatical leave at Instituto Nacional de Astrofisica, Optica y Electrónica, Tonantzintla, Puebla, México*

*Corresponding author e-mail: [*asosa@ifuap.buap.mx*](mailto:asosa@ifuap.buap.mx)


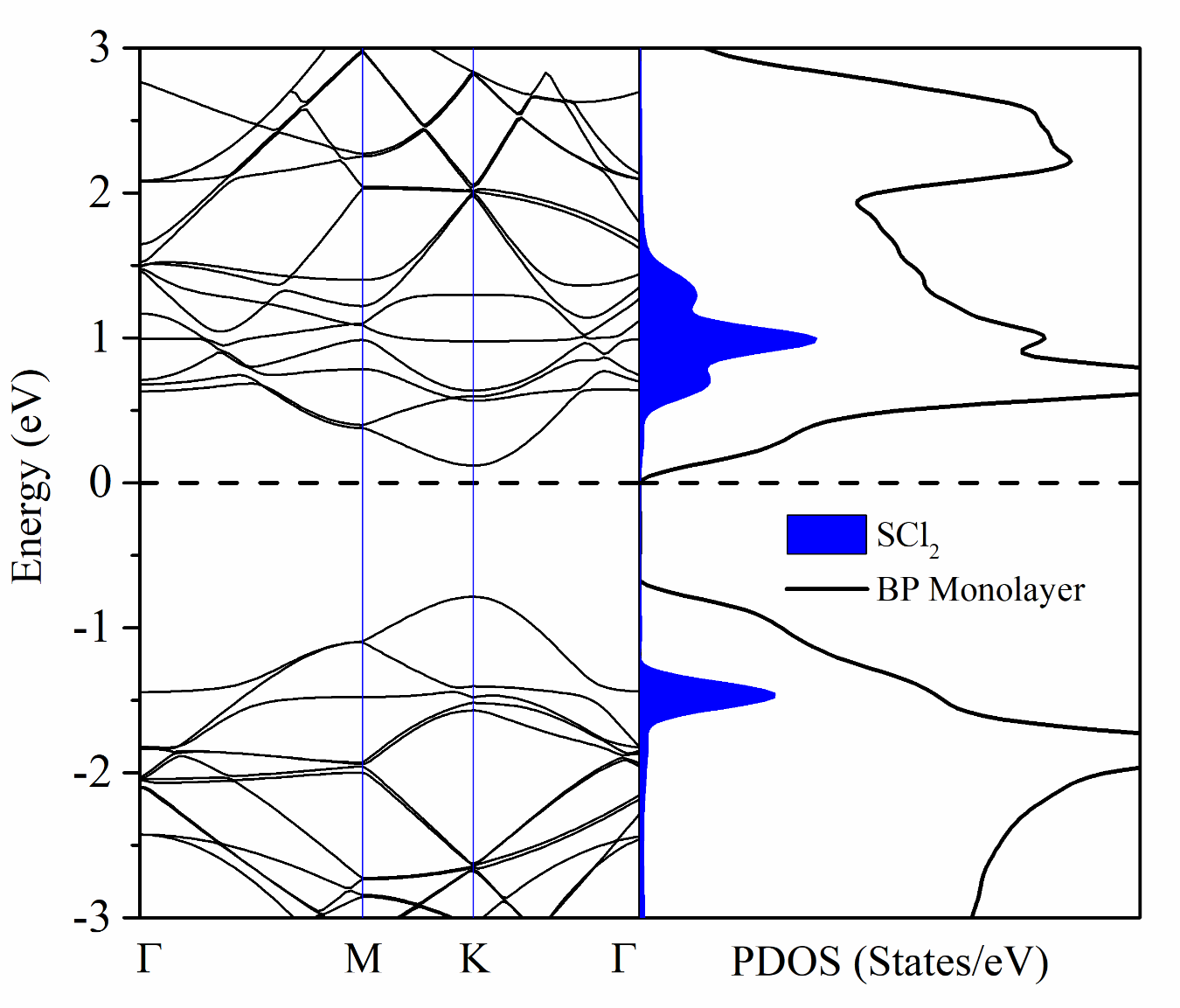


**Fig. S1** Band structures and PDOS of SCl_2_ molecule adsorbed on the BP monolayer surface

**
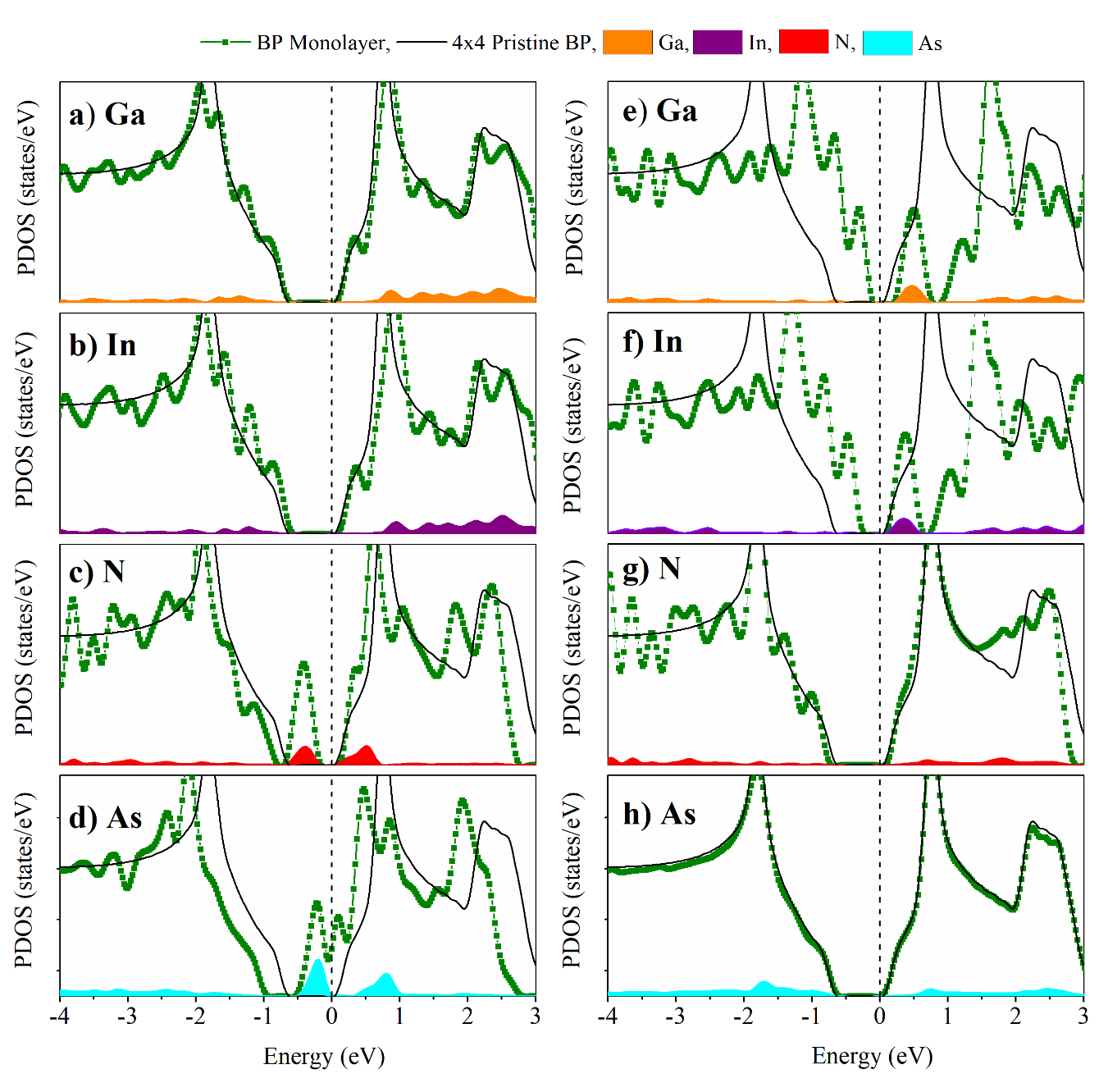
**

**Fig. S2** PDOS of metal-doped BP monolayer systems a) Ga, b) In, c) N and d) As for the substituted B atom, e) Ga, f) In, G) N and h) As for the substituted P atom


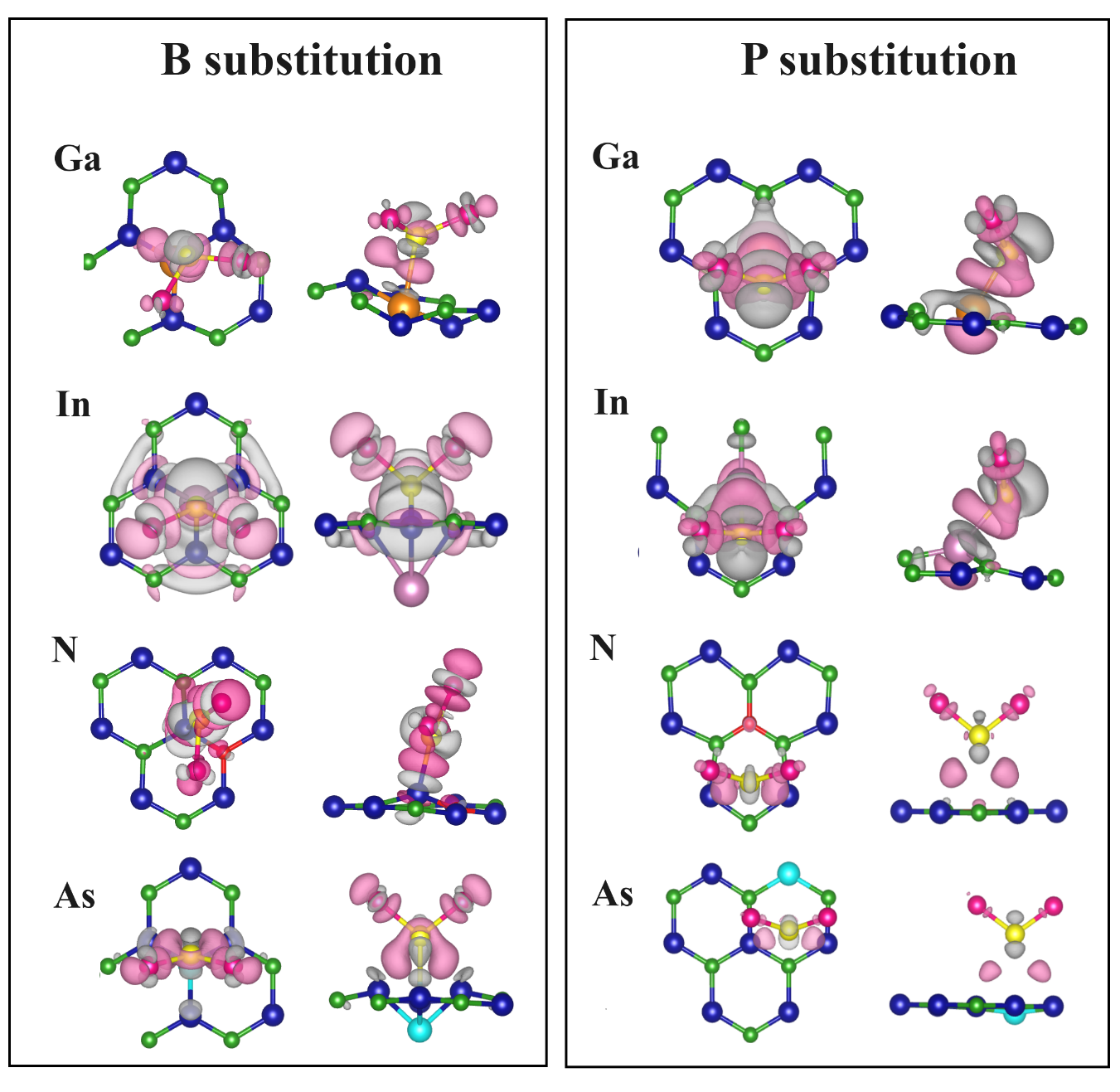


**Fig. S3** Charge density difference (CDD) isosurfaces for the representative SCl_2_ metal-doped BP monolayer system. The left and right schemes represent the B substitution y P substitution, respectively. Pink and grey regions refer to electron accumulation and depletion, respectively


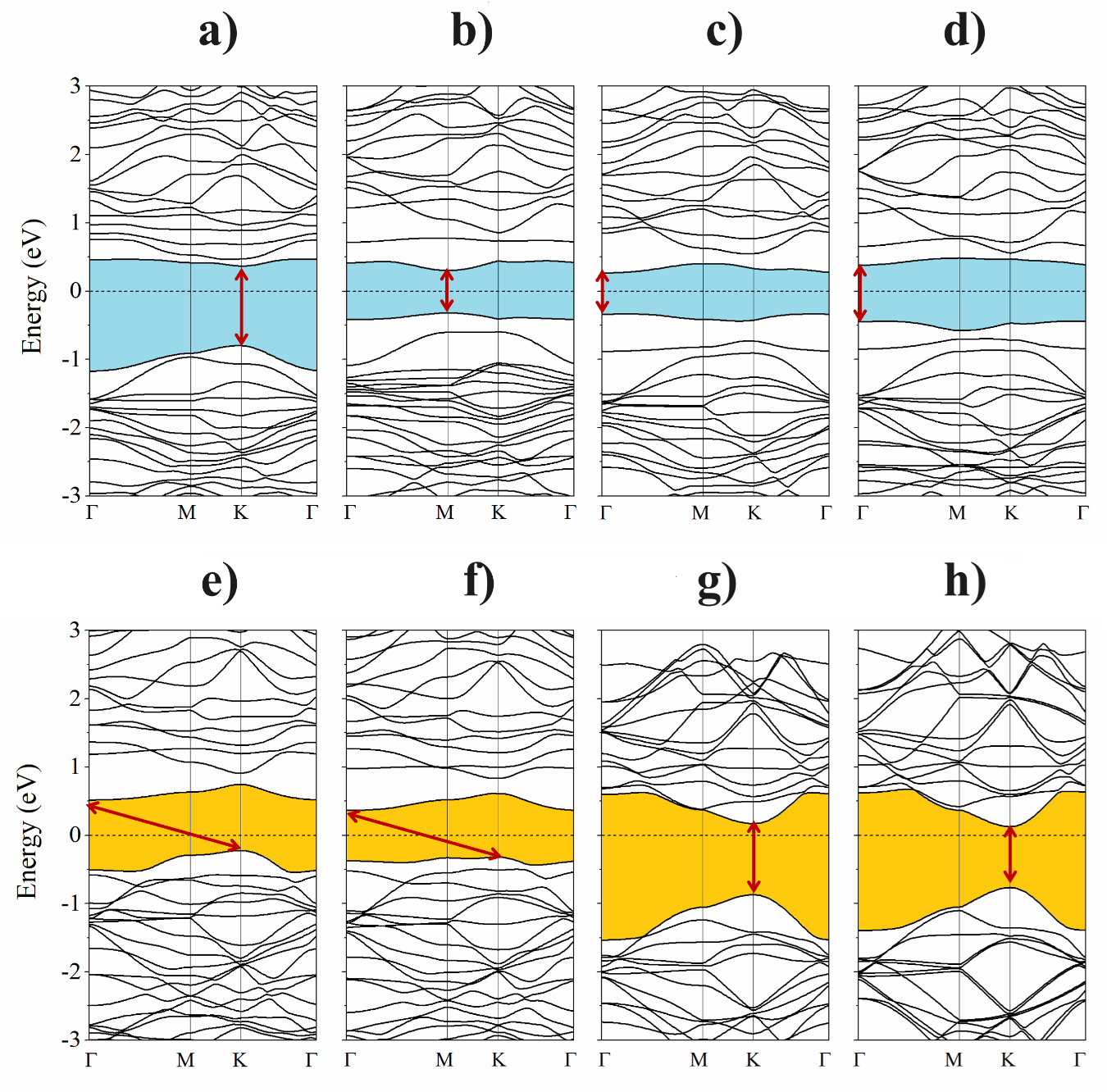


**Fig. S4** Electronic band structures of the SCl_2_ adsorbed on the metal-doped BP monolayer, a) Ga, b) In, c) N and d) As for the substituted B atom, and e) Ga, f) In, G) N and h) As for the substituted P atom

**Table S1** Bond angle information before and after the optimization of the molecule in the structure

| System | Bond angle | | | |
| --- | --- | --- | --- | --- |
| Molecule (SCl_2_) | 104.99º |  |  |  |
| BP ML+ SCl_2_ | 104.23º |  |  |  |
| Doped BP ML+SCl_2_ | Ga-SCl_2_ | In-SCl_2_ | N-SCl_2_ | As-SCl_2_ |
| B | 104.38º | 91.17º | 96.86º | 100.19º |
|  |  |  |  |  |
| P | 102.43º | 103.93º | 103.26º | 103.99º |
|  |  |  |  |  |


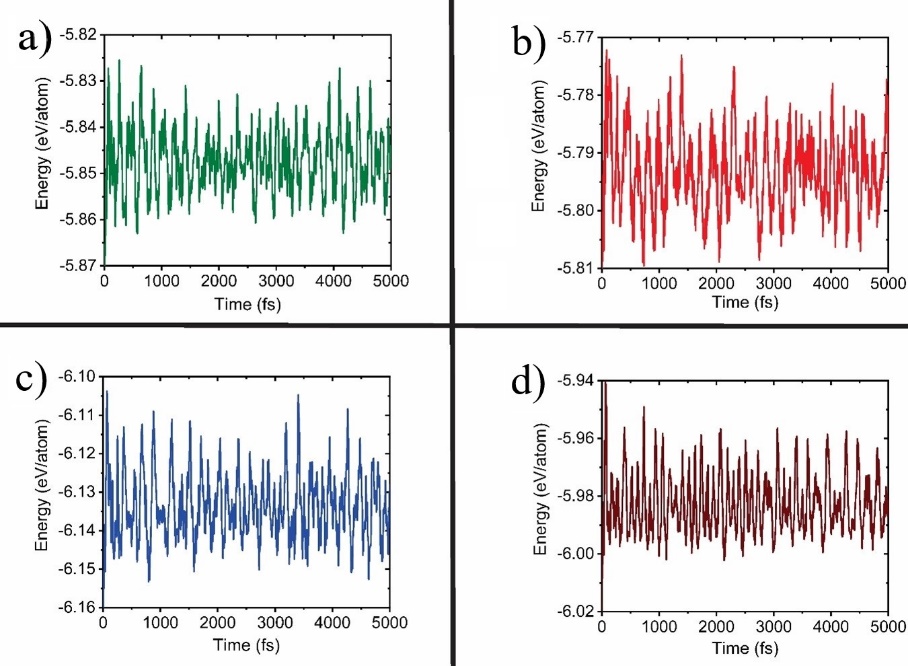


**Fig. S5** The MD simulation energies to examine the stability of metal-doped BP ML system at 300 K, for the B substituted by: a) Ga, b) In, c) N and d) As atoms
